# A Homogeneous Breast Phantom Measurement System with an Improved Modified Microwave Imaging Antenna Sensor

**DOI:** 10.3390/s18092962

**Published:** 2018-09-05

**Authors:** Mohammad Tariqul Islam, Md. Samsuzzaman, Md. Tarikul Islam, Salehin Kibria, Mandeep Jit Singh

**Affiliations:** Centre of Advanced Electronic and Communication Engineering, Faculty of Engineering and Built Environment, Universiti Kebangsaan Malaysia, Bangi 43600, Selangor, Malaysia; kibriasalehin@gmail.com (S.K.); mandeep@ukm.edu.my (M.J.S.)

**Keywords:** homogenous breast phantom, antipodal Vivaldi antenna, microwave imaging, breast tumor detection

## Abstract

Microwave breast imaging has been reported as having the most potential to become an alternative or additional tool to the existing X-ray mammography technique for detecting breast tumors. Microwave antenna sensor performance plays a significant role in microwave imaging system applications because the image quality is mostly affected by the microwave antenna sensor array properties like the number of antenna sensors in the array and the size of the antenna sensors. In this paper, a new system for successful early detection of a breast tumor using a balanced slotted antipodal Vivaldi Antenna (BSAVA) sensor is presented. The designed antenna sensor has an overall dimension of 0.401λ × 0.401λ × 0.016λ at the first resonant frequency and operates between 3.01 to 11 GHz under 10 dB. The radiating fins are modified by etching three slots on both fins which increases the operating bandwidth, directionality of radiation pattern, gain and efficiency. The antenna sensor performance of both the frequency domain and time domain scenarios and high-fidelity factor with NFD is also investigated. The antenna sensor can send and receive short electromagnetic pulses in the near field with low loss, little distortion and highly directionality. A realistic homogenous breast phantom is fabricated, and a breast phantom measurement system is developed where a two antennas sensor is placed on the breast model rotated by a mechanical scanner. The tumor response was investigated by analyzing the backscattering signals and successful image construction proves that the proposed microwave antenna sensor can be a suitable candidate for a high-resolution microwave breast imaging system.

## 1. Introduction

Recently, early detection of breast cancer has been identified as a vital preventative measure against untimely deaths among women all over the world. The main cause is the presence of one or more malignant cell clusters inside the breast tissue [1]. The survival rate can be increased up to 97% by early detection and treatment but for this, a highly reliable system for early detection with an exceedingly efficient method is required [2]. Healthcare diagnosis tools based on microwaves have been prioritized by researchers these days. The contrast of microwave signals scattered from different tissue types can be distinguished by microwave antenna sensors. Radiated and scattered power are received by one or more antenna sensors in microwave imaging. Microwave-based portable medical diagnosis tools can save lives by utilizing microwave sensor antennas that have high efficiency and sensitivity. In this regard, a microwave imaging sensor could be an alternative tool to existing conventional approaches like X-ray mammography, MRI, and ultrasound imaging for early detection of breast tumors [3].

The main benefits of microwave imaging systems are their low complexity, low cost, and the fact they do not produce ionizing radiation. Microwave imaging technology has made rapid progress in recent years, but it is still a challenge to produce an accurate and high-resolution image of the scattered signals. To date, numerous broadband and planar printed monopole microwave antennas have been developed for breast cancer detection due to their simple structure, broadband property, compact size, and ease of fabrication. The antenna used as transceiver should be compact, wider bandwidth and high gain for an efficient microwave imaging system with high resolution and dynamic range [4]. The main working principle is based on the difference between the dielectric properties of healthy breast tissue and malignant cells. The water content of every biological tissue varies, which causes different electrical properties. Furthermore, the presence of ions and free radicals increase the dielectric loss in malignant tissues. Thus, the tumor, with higher dielectric constant than normal breast tissue, can be detected by analyzing the scattering signals from single or multiple illuminations [5]. Several types of antennas are developed for breast phantom measurements, such as the unit cell antenna [6], the cross-Vivaldi antenna [7], the compact metamaterials antenna [8], and the slot antenna [9,10]. In this case, the antipodal Vivaldi antenna can be a good candidate for its high directive radiation patterns, compact size and higher gain [11]. The challenge of designing a Vivaldi antenna is how to obtain a directional radiation pattern and resonance at a lower frequency. Vivaldi antenna sensors in medical applications have been the subject of research for the last few years [12]. Various researchers have proposed different techniques to enhance the radiation performance of Vivaldi antennas at higher frequencies [13]. Different techniques including the use of lenses [14], zero index material [15], high permittivity material were applied to lead the energy in the aperture flare in the end-fire direction. One of the methods of enhancing the performance of the Vivaldi antenna is to add a parasitic ellipse into the patch [16]. It expands the field coupling but the size is not compact (140 × 66 mm^2^) and antenna fails to operate in lower frequencies. In [17], a tapered slot square (75 × 75 mm^2^) shape Vivaldi antenna was proposed. The antenna has a directional radiation pattern but fails to achieve higher frequencies. A cavity-backed Vivaldi antenna for breast phantom screening was proposed [5], where the size of the antenna is compact but the gain is not satisfactory. Investigations reported in [18] showed that an improved Vivaldi antenna was anticipated with planar directors at front of the transverse resonator and aperture for increasing the directivity and gain but the dimensions were increased dramatically and VSWR was not contagious. An antipodal Vivaldi antenna with compact size was reported in [19] which achieves a good operating bandwidth but the efficiency and gain were less than 70% and 5 dBi, respectively. The directivity of the Vivaldi antenna reported in [20] was improved, but the size exceeds the acceptable dimensions for use in portable devices. A compact antipodal Vivaldi antenna was reported in [21] where the antenna achieves wide bandwidth, but the gain is low at the lower frequency band and its structure is complex. An Exponential Slot Edge (ESE) antenna was proposed in [22] where a palm tree antipodal Vivaldi structure was used to achieve directional radiation, but the antenna fails to attain lower frequency band and higher gain. A fern leaf type fractal structure was introduced to achieve Vivaldi properties but there is no clarification of microwave imaging technique and in addition no simulation or measurement results were presented [23]. An antenna array with 12 grooved slot components was used for an ultra-wideband (UWB)-based microwave imaging system that achieves acceptable gain [24]. The array produced a 5 GHz band that makes the antenna unusable over the lower portion of the UWB bandwidth. Several numbers of antennas with different frequencies, dimension, gain, and efficiencies have been proposed for microwave imaging systems. The system proposed in [25] implemented microwave imaging but the system is expensive and not compact. A new handheld tumor detection system was proposed in [26], where a cross-shaped array was designed covering human breast but the system is complex and the image constructed is noisy and it is hard to differentiate the tumor presence from the post-processing alone. Several researchers have reported numerous time-domain measurements using breast imaging systems [27,28,29,30]. The main advantages of time domain measurements are less screening time and cost-effectiveness. A multistatic radar-based experimental system with a 16-unit antenna array for the detection of breast cancer was proposed in [27]. A time-domain system for microwave breast cancer detection was proposed in [29] where an antenna array was tested with a phantom but the antenna array failed to attain the lower band and no imaging results were classified. Kwon et al. proposed a time-domain breast cancer imaging system based on CMOS circuits, which is both quick and cost-effective. The low signal-to-noise ratio (SNR), which is an inherent drawback of time domain systems, can be significantly improved by the signal averaging of multiple measurements [31]. However, the signal averaging of repeated measurements introduces signal distortions due to clock jitter. Gaussian bandpass filtering (BPF) for image filtering was used in [30] for time domain microwave imaging of breast cancer. Researchers at McGill University studied the time-domain radar-based imaging for breast health intensive care [32,33]. The system used a very high-speed oscilloscope, high-precision pulse generator and complex switches. It requires a very quick sampling clock in a way that even a little jitter inside the sampling clock can blur the images [34]. The problem of time domain measurement is a low signal to noise ratio resulting with the high frequency radio frequency signal reduces drastically inside breast tissue. Therefore, the signal should be measured frequently at spatially diverse points around the region of interest to improve SNR, as the SNR is relative to the square root of the number of measurements.

In this paper, an improved BSAVA with enhanced UWB band for microwave breast imaging sensor is presented. The fins are etched with three slots on each side to enhance the bandwidth and gain as well as the directive radiation pattern that is coherent with the size of the antenna. The antenna sensor operates within 3.1 to 11 GHz with an evenly distributed current that helps to achieve a highly directive radiation pattern and a good gain of 6.8 dBi. Simulated and measured results of both frequency domain and time domain are also presented to validate the use of the antenna sensor in a microwave imaging system. The antenna sensor performance is tested by developing a realistic breast phantom screening system for detecting malignant cells inside the breast. The authors used a cylindrical mechanical setup where two antenna sensors are placed at a distance of 255 mm at opposite sides of the cylinder and rotated 360° around the phantom with a stepper motor controlled by an Arduino-Uno module. The transmitting and receiving antennas are directly connected to a PNA Network Analyzer. The main objective of the screening system is to track the behavior of the change of backscattering signals received from the receiver antenna sensor. This allows identifying the change of dielectric properties of the different elements of the breast phantom. The noticeable change in received signals is the main issue in identifying the tumor. Further analysis of the signals concludes with the decision whether the system can be effective for detecting tumors inside a human breast.

## 2. Antenna Sensor Design Structure

**Figure 1:**
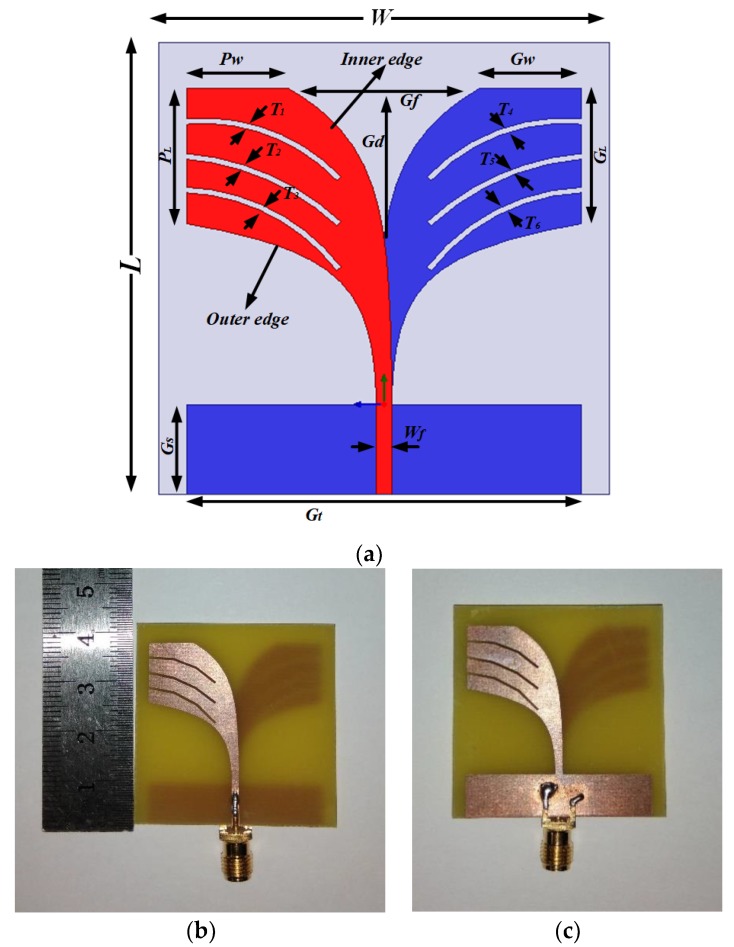
(**a**) Antenna Sensor geometry; fabricated prototype (**b**) front view and (**c**) back view.

The main aim of this design was to identify the differences in dielectric properties between healthy breast tissue and tumorous tissues. Multiple resonance frequencies are necessary for better penetration and high-resolution imaging in the deeper portion of the breast [5]. The prerequisite for microwave imaging is that the antenna sensor must have high gain, a directive radiation pattern, wider bandwidth and lower resonance frequency. The most promising candidate for all this is the Vivaldi antenna sensor for its higher gain, and highly directive radiation due to its high peak value for pulse coverage. It offers a narrow pulse width with a stable group delay. A conventional Vivaldi antenna is modified to achieve this characteristic by introducing BSAVA [22,23,35,36]. Figure 1 shows the geometric layout and fabricated prototype of the proposed BSAVA. The antenna sensor is printed on the low-cost FR-4 substrate with a thickness of 1.6 mm, loss tangent of 0.02 and relative permittivity of 4.4. The overall dimension of the BSAVA is 40 × 40 × 1.6 mm^3^. The main radiating element is determined by modified patch and ground with curved slot line, radiating fins and the feeding line. These parameters are adjusted to get desired antenna specification by etching the slots. Asymmetrical slots are examined for increasing the electrical length to decrease the lower band. The inner and outer edge of the tapered shape is estimated by:

(1) $$x_{i}=\pm c_{s}\cdot \exp(k_{s}y)\mp (c_{s}+0.5\cdot c_{w})\;$$

(2) $$x_{0}=\pm c_{w}\cdot \exp(k_{w}y^{sf})\mp (c_{s}+0.5\cdot c_{w})\;$$

where *x*_i_ and *x*_0_ both show the distances from the slot centerline towards the inner and outer edges, individually [37]. The cutoff frequency of the proposed prototype is estimated by the equation reported in [38]:

(3) $$f_{r}=\frac{c}{\left[w^{\prime }\sqrt{\left(\varepsilon_{r}\right)}\right]}\;$$

where *f_r_* is resonant frequency, *c* is the speed of light, *w^′^* is the width of the opening blaze edge and $\varepsilon_{r}$ is the relative permittivity of the prototype material. Three 0.5 mm width slots have been cut in each fin of both the top and bottom radiator to achieve larger bandwidth. These narrow cuts have the significant effect of surface current distribution which helps to enhance the lower band. For balancing the Vivaldi antenna, a partial slot at the bottom of the ground plane with the height of *G_s_* has been cut out. The antenna sensor is fed with a 50 Ω SMA connector which has a dielectric constant of 2.08 and electrical conductivity of 4.62 × 104 S/m. Different adjusted design parameters are presented in Table 1.

**Figure 2:**
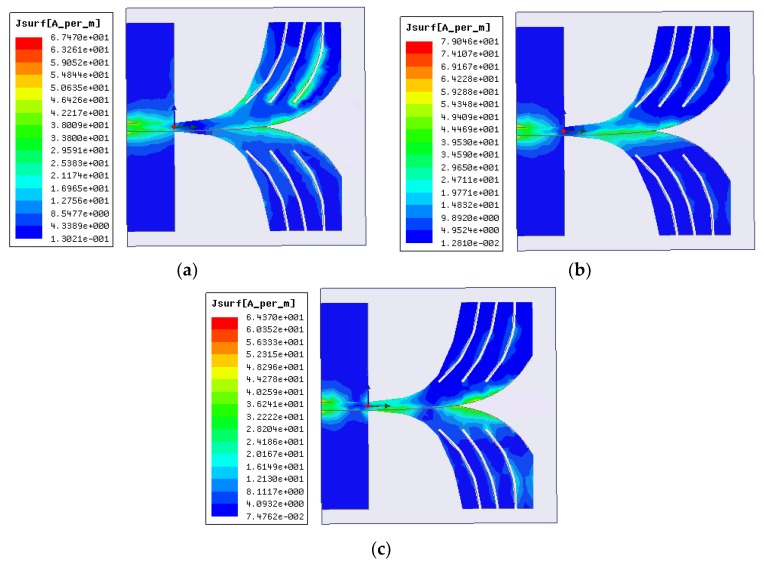
Simulated surface current distributions at (**a**) 3.28 GHz, (**b**) 4.24 GHz, and (**c**) 6.16 GHz.

The surface current distribution of the proposed BSAVA for different frequencies of 3.28, 4.24 and 6.16 GHz is shown in Figure 2. Most of the current is conducted through the radiating fins and around the cutting slots. The current is well distributed along with the patch and ground at the lower frequencies. Several nulls are observed at the higher frequencies because of the higher-order current mode excitation. The current paths are changing due to the slots which produce higher order current mode. This characteristic has a great effect on antenna performance. Simultaneously, the gain in the main lobe has improved and radiation pattern improves remarkably. The slots help to avoid tapping, as a result, the control over current distribution is restored to the adjacent edges of the BSAVA.

## 3. Parametric Study

**Figure 3:**
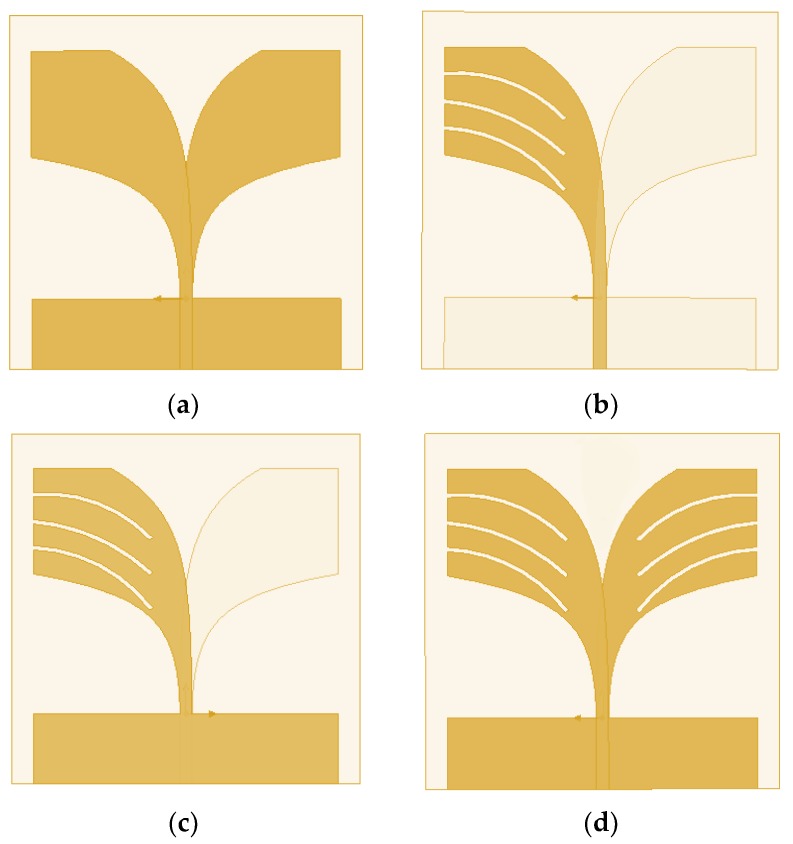
Different modifications on patch and ground (**a**) basic (**b**) patch etched (**c**) ground etched (**d**) proposed shape.

**Figure 4:**
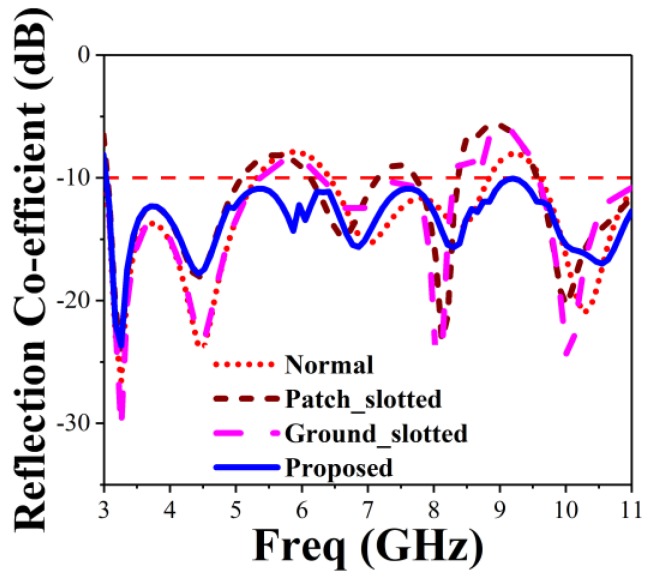
Simulated reflection coefficient for different modification on patch and ground.

**Figure 5:**
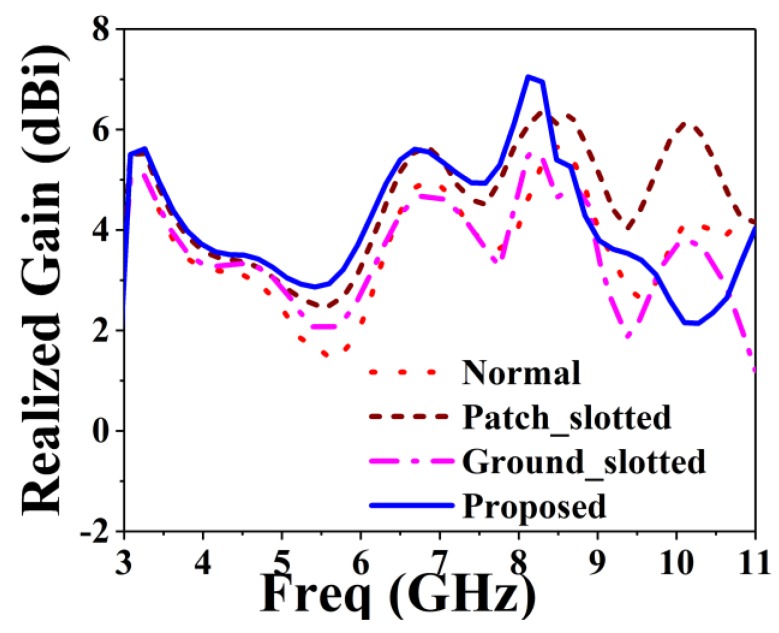
Simulated peak realized gain for different modifications.

The proposed BSAVA sensor comprise a patch and ground facing 180° with respect to each other. A rectangle-shaped plane attached to the ground with the length and width denoted by G_s_ and G_t_ for balancing the Vivaldi antenna. Three slots each has been cut out from the radiating fins of the antenna denoted by *T*_1_, *T*_2_, *T*_3_, *T*_4_, *T*_5_, and *T*_6_. There is a significant effect of changing the width of the fins on antenna performance. Figure 3 denotes different adjustments on patch and ground of the basic BSAVA sensor. First of all, the slots etched from both patch and ground has the same size. Afterward, the antenna parameters are being investigated. Finally, satisfactory results are obtained with the modifications which exhibit the desired features of the BSAVA. Table 2 represents the evaluations of the effects of different modification of antenna design on performance. Different modifications including the final design are illustrated in Figure 4 and Figure 5. The observation shows that the proposed prototype has wider bandwidth comparing to the other tested shapes including basic, patch slotted and ground slotted designs. The basic BSAVA has a bandwidth of 3.01 to 5.31 GHz under 10 dB. By applying slots in the patch, the starting frequency has shifted to a lower frequency of 2.90 GHz but the bandwidth is 2.90 to 5.12 GHz. Again, by using the slot in the ground the bandwidth is slightly increased to upper frequency from 3.01 to 5.36 GHz but it doesn’t cover the UWB band. In contrast, in the proposed BSAVA with six slots in the patch and ground the lower frequency started from 3.01 GHz and the higher frequency is significantly shifted to 11 GHz with a continuous wideband frequency that covers the entire UWB frequency band. The peak gains for the tested shapes are also presented in Table 2.

The proposed antenna reached a peak gain of 7.06 dBi, while the basic, patch slotted and ground slotted BSAVA has the gain of 5.62, 6.33 and 5.54 dBi, respectively. At the lower frequency, the gain is increased due to the modification of the radiating elements. The slots in both the radiator increase the electrical length that create strong directional radiation because of the mitigation of the surface current in the vertical direction which does not contribute to radiation in the end-fire direction. This modification has a significant effect on gain and efficiency.

## 4. Antenna Sensor Performance Measurement

**Figure 6:**
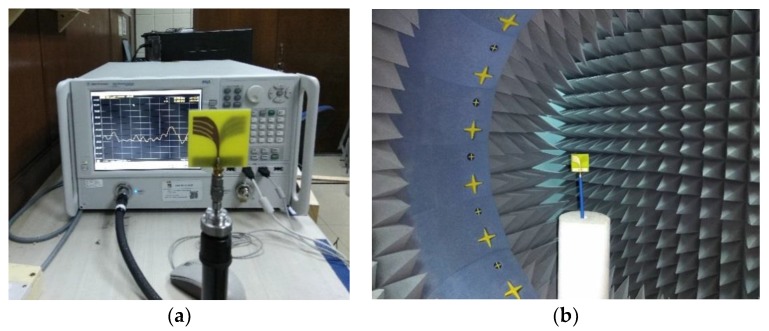
(**a**) PNA measurement setup (**b**) Satimo StarLab setup.

The performance of the proposed prototype has been analyzed and adjusted by using the CST microwave studio (Dassault Systèmes SE, Vélizy-Villacoublay, France). The data analysis and scientific graphing software Origin Pro (OriginLab Corporatio, Wellesley Hills, MA, USA.) is used to plot the simulated results. A PNA series vector network analyzer (E8362C 10 MHz-67 GHz) from Keysight Technologies (Santa Rosa, CA, USA) is used to measure the S_11_ responses and the setup is shown in Figure 6a. A StarLab near-field antenna measurement system (Microwave Vision Group, Paris, French) as shown in Figure 6b is used to measure the radiation pattern, efficiency, and gain of the prototype. The system measures the radiated power of the antenna in the near field region for computing the equivalent far-field values of the antenna under test (AUT). The AUT is positioned in the middle of a circular arch on the test bed which consists of 16 separate receiving antennas. The antennas are evenly spread out across the arch. The AUT is rotated 360 degrees horizontally to create a full 3D scan from which we get the 3D radiation pattern. The gain and efficiency are computed from the far field data.

### 4.1. Frequency Domain Performance

**Figure 7:**
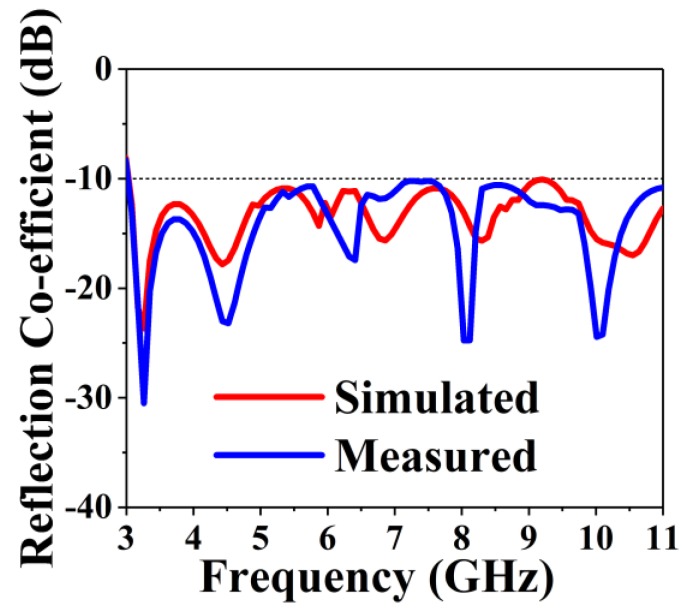
Simulated and measured reflection coefficient over the frequency.

The measured and simulated reflection coefficient (S_11_) curves of the optimized design are displayed in Figure 7. The BSAVA has an operating bandwidth of 7.9 GHz ranging from 3.01 to 11 GHz. The starting resonance frequency is 3.01 and the highest resonance frequency is noticed at 3.2 with several peaks across the bandwidth. The modified slot affects the BSAVA to acquire the lower frequency bandwidth. A good agreement is observed between the measured and simulated results. Being compact and simple design, the proposed prototype has a wider bandwidth than other recently reported antennas [16,17,39]. The entire bandwidth has no band gap.

**Figure 8:**
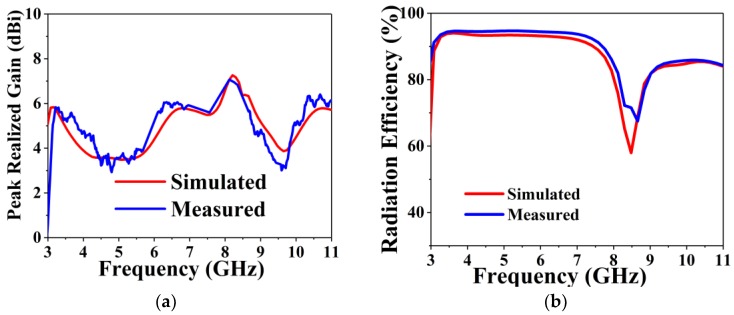
Simulated and measured (**a**) peak realized the gain and (**b**) radiation efficiency over frequency.

**Figure 9:**
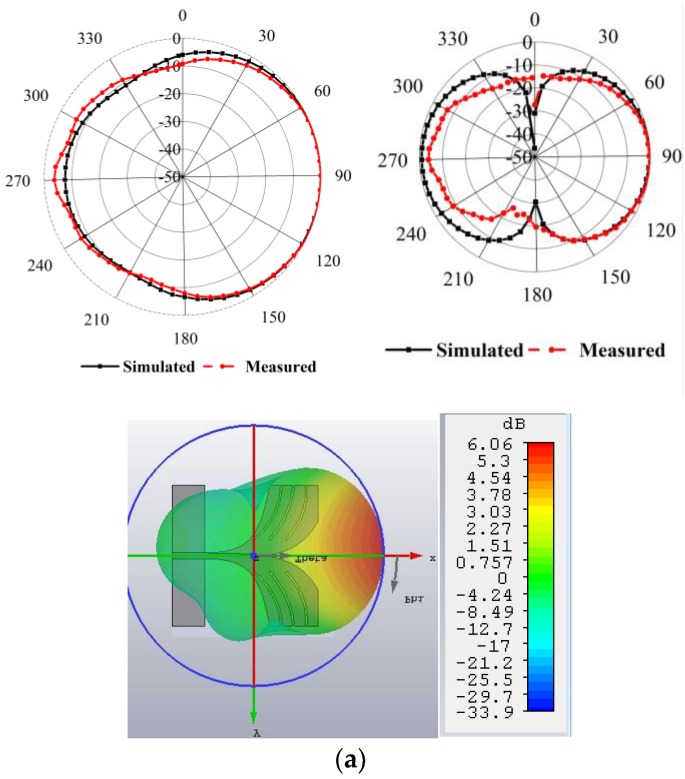

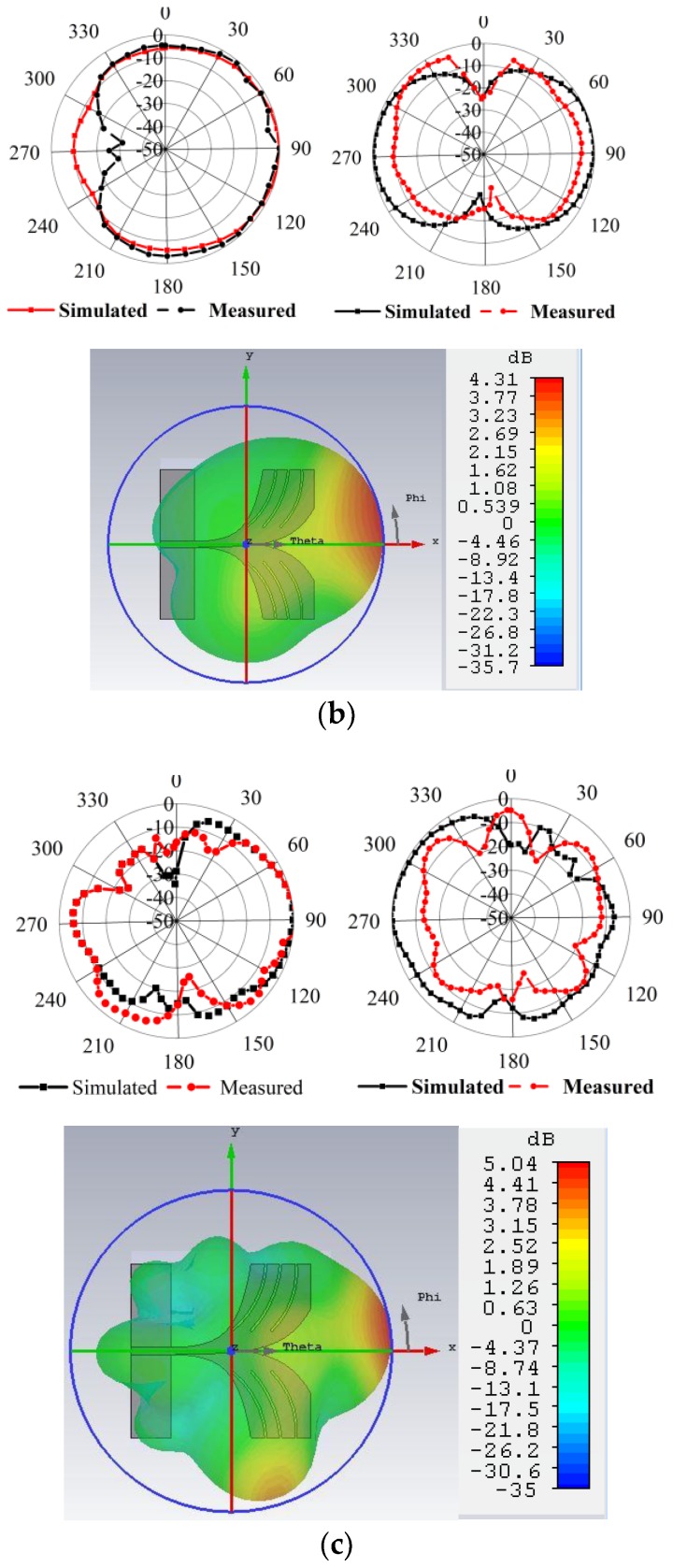
Simulated and measured 2D and 3D radiation patterns at different frequencies, (**a**) 3.28 GHz (**b**) 4.24 GHz (**c**) 6.16 GHz.

The measured and simulated peak gain against frequency is shown in Figure 8a. The BSAVA has the higher gain at the lower frequency band which is essential for microwave imaging systems. The average peak gain is 6 dBi along with the maximum peak gain of 7.1 dBi at 8.2 GHz. The simulated and measured results are consistent. The BSAVA achieves a better gain being simple and compact structure compared to recently published Vivaldi antennas. Figure 8b displays the measured and simulated radiation efficiency of the proposed BSAVA with respect to frequency. From the graph, we can see that the average radiation efficiency is about 92% with a maximum of 95%. A good agreement between the simulated and measured results was observed. The measured and simulated 2D and 3D radiation patterns are illustrated in Figure 9 for three different resonance frequencies of 3.28, 4.24 and 6.16 GHz. The Phi and Theta spherical coordinates are related to the Cartesian axis’s configurations such as Theta = 0° to 360° is the XZ cut whereas Phi = 0°, Theta = 0° to 360° and Phi = 90° is the YZ cut and Theta = 90°, Phi = 0° to 360° is considered the XY cut. The XZ-plane (ϕ = 0°) is considered as E-plane and YZ-plane (ϕ = 90°) is considered as H-plane. From the near field measurement, the proposed BSAVA is directional and the main radiation direction is towards the boresight. The key lobes of the radiation patterns are fixed towards the end-fire direction over the entire frequency band. The BSAVA has a stable radiation pattern that guarantees a higher rate of scattered signals having low noise density at the backward direction. With the increase of frequency antenna directivity is increased with the higher order modes.

### 4.2. Time Domain Performance

**Figure A1:**
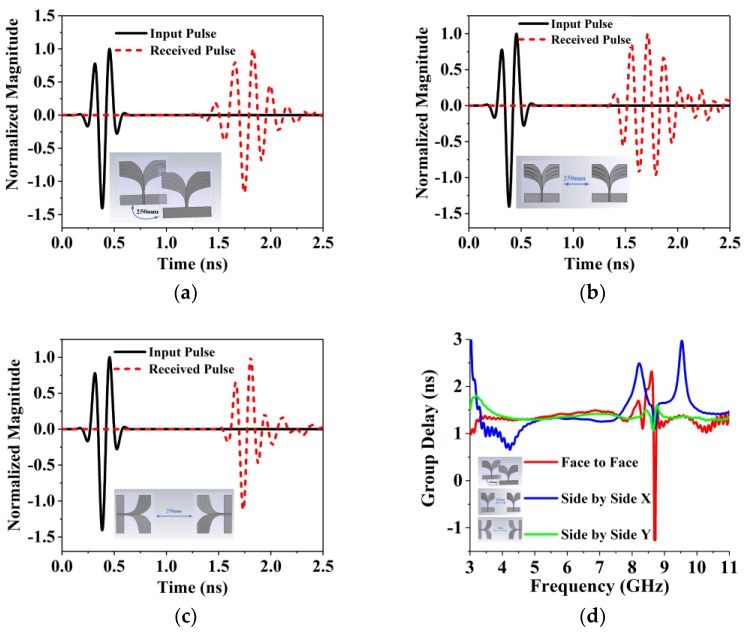
Normalized magnitude of three different setup (**a**) face to face; (**b**) side by side X; (**c**) side by side Y and (**d**) group delay.

Appendix A examines the performance of the time domain performance of the BSAVA at three different scenarios of face to face, side by side X and side by side Y at 250 mm distant is presented in Figure A1a–c. To validate the correspondence between the transmitter (TX) and received (RX) signals, the fidelity factor needs to be determined. The fidelity factor is calculated at 0.94, 0.85 and 0.98 using Equation (A1) for the face to face, side by side X and side by side Y position, respectively. The group delay is measured from the negative derivatives of the phase response against frequency and presented in Figure A1d. Among the three set ups the group delay of side by side, Y is less than both the face to face and side by side X. This is almost constant over the frequency, as a result, this setup is recommended for microwave imaging systems. In Appendix B, the quality factor and Near Field Directivity (NFD) are also calculated and presented. To verify the physical dimensions of the designed antenna, we have also analyzed the theoretical and calculated Q factor. In order to investigate the amount of power coupled to the tissue, we computed a near-field directivity (NFD) factor of about 60% for the antenna.

## 5. Homogenous Phantom Development and Measurement

**Figure 10:**
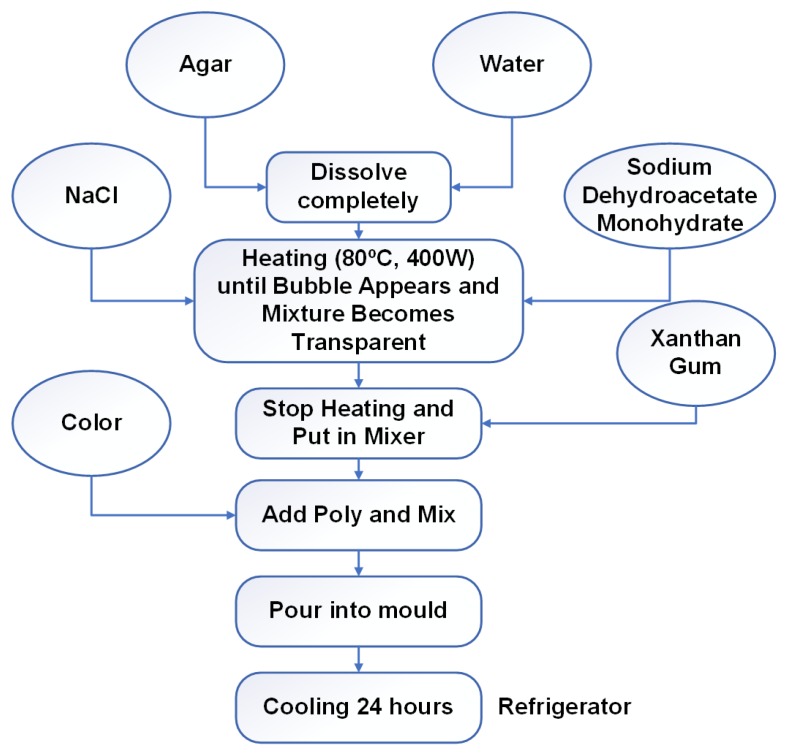
An outline of the homogeneous phantom fabrication.

**Figure 11:**
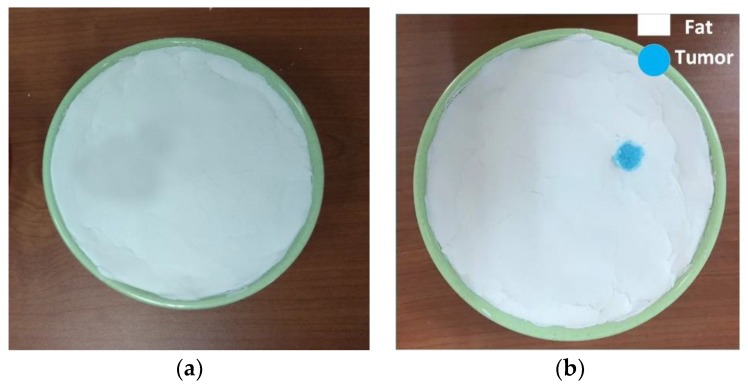
Fabricated homogenous breast phantom, (**a**) without tumor and (**b**) with a single tumor inside the phantom.

In the construction of a homogeneous phantom, the breast was considered as one whole fatty layer with a tumor inside. The materials and mixing ratios for the homogeneous phantom, tumor, and skin are listed in Table 3 below. For the homogenous phantom, sodium chloride (NaCl), polyethylene powder, agar powder, xanthan gum, sodium dehydroacetate monohydrate and distilled water were used. Polyethylene powder was used to adjust the permittivity and NaCl to adjust the conductivity. Agar was used to maintain the shape of the phantom by preventing separation of water content, xanthan gum was used as a thickener and sodium dehydroacetate monohydrate as a preservative. Basically, the materials which alter the dielectric properties of this method are NaCl, polyethylene powder, agar and distilled water, and they are the main ingredients in the phantom. The procedure of the phantom fabrication for each layer is represented by a flowchart in Figure 10. The fabricated homogenous phantoms which have two layers of fat and tumor without a tumor and with a single tumor are displayed in Figure 11a,b, respectively, and were developed in the UKM organic lab. The radius of the phantom is 50 mm and the tumor is located at 25 mm distance from the center of the phantom. The tumor diameter and height are 10 mm and 40 mm, respectively. The measurement is taken within the frequency range of 3.1–10.6 GHz. The measurement of the dielectric constant (Dk) and loss tangent (Tan δ) are conducted by using a dielectric probe from a KEYSIGHT 85070E Dielectric Probe Kit with the 85070 software installed with a PNA-L N5232A 300 KHz to 20 GHz vector network analyzer (VNA, Keysight Technologies, Santa Rosa, CA, USA). Measurements of the dielectric constant (Dk) and loss tangent (Tan δ) are conducted. The probe test is indicated as one of the most popular and easy to use methods to obtain the dielectric properties [40,41,42], and it is considered as a non-destructive test that doesn’t affect the measuring substrate with a probe method measurement capability of up to 20 GHz [43]. 

The testing process starts by doing the open, short and 25 ccs sterile water calibration procedure. The setting of the dielectric probe measurement system distributed with the Keysight Dielectric Probe model 85070E connected to the performance network analyzer (PNA-L) model N5232A up to 20 GHz via a high-temperature coaxial cable. The dielectric constant of fat is 12.58 and the conductivity is 0.141 S/m and the tumor has a dielectric constant of 57.37. The tumor has a high dielectric constant due to its higher water content [44]. We have also prepared a 3 mm skin layer and the measured dielectric properties of the skin layer material are 23.44. The measured dielectric constant of the muscle-like material which has been placed under the breast phantom to analyze the body effect is near about 44. The higher the frequency, the higher is the attenuation and vice versa. The lower the frequency, the larger the wavelength, and the deeper the penetration power. Penetration is not just about energy, it is also about the properties of the material and the various modes of interaction between electromagnetic radiation and matter. Each material has an absorption and reflection spectrum because of this penetration. Low-frequency EM radiation is highly penetrating as the photons do not have enough energy to be absorbed by atomic transitions, molecular resonances and so on. For some tissues, at higher frequencies or shorter wavelengths, some of these modes of interaction become possible. It depends on skin depth which has an inverse relationship with conductivity and frequency. In general, EM penetration into a lossy material has an inverse dependence with frequency. The penetration depth (or better skin depth) depends on the dielectric and sometimes magnetic properties of the medium. Important values are e″ and µ″ which are the dielectric or magnetic loss factors. In plain words the higher those values are the more energy they take out of the field, reducing the amplitude and therefore its penetration depth (like extinction coefficients in optics). Those loss factors are not constant over frequency. Often e″ increases with increasing frequency, enhancing absorption and reducing penetration.

**Figure 12:**
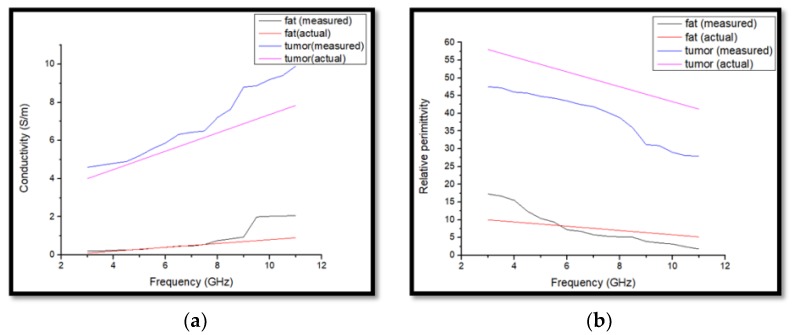
(**a**) Conductivity and (**b**) relative permittivity of the phantom.

Figure 12a,b shows the measured results with a standard curve of the conductivity and relative permittivity of the homogeneous breast phantom. From Figure 12, it can be seen that after increasing the frequency, fat and tumor relative permittivity decreases but conductivity increases.

## 6. Microwave Imaging System Setup

**Figure 13:**
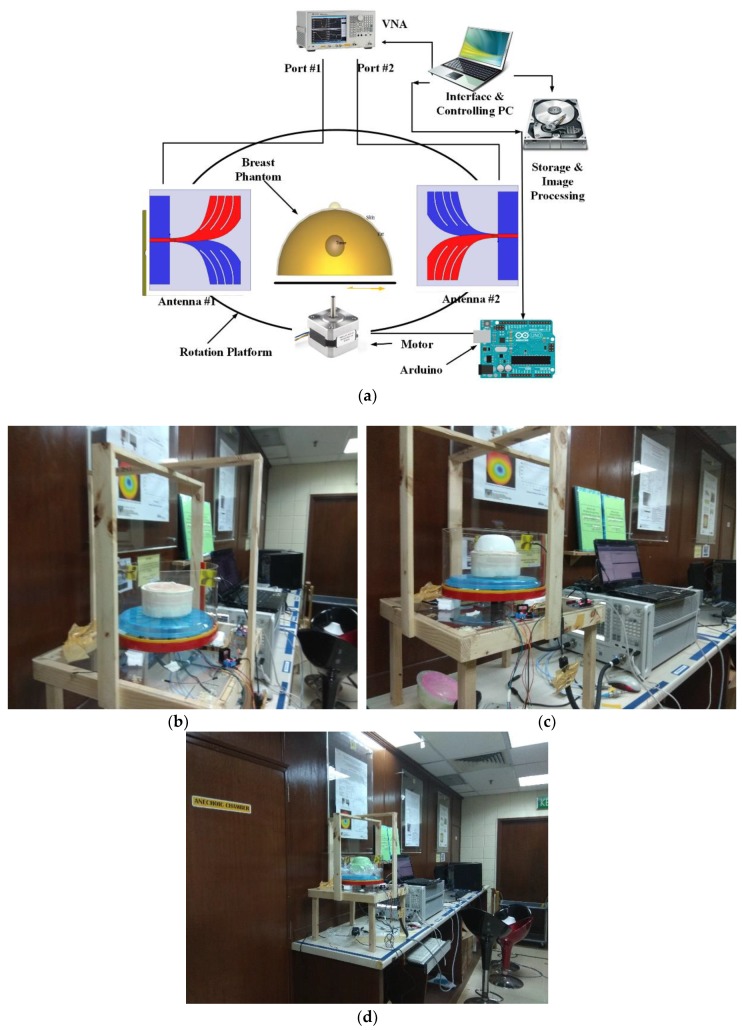
(**a**) Microwave imaging system block diagram, (**b**) Experiment setup of cylindrical container filled with the muscle material under breast phantom (**c**) Experiment setup without the skin layer of the phantom and (**d**) Experiment setup with a skin layer of surrounding Phantom to observe the effect of skin layer.

The main aim of the imaging system is to identify the change in backscattering signal with the presence of different dielectric properties of human breast tissue including the high dielectric tumor. An automated system is developed where all the mechanical parts are controlled by a single PC, the antenna sensor is working as a transceiver and the signals are collected by a PNA. The block diagram and practical measurement setup of the full imaging system are shown in Figure 13a–d. The data is collected from a mechanical setup of a rotating platform where an Arduino Uno and stepper motor driver are used to control the rotation of the system from 0 to 2π. 360° rotation was performed where each step was 7.2° total counting 50 points. For testing, the phantom is rotated and we collect the data to identify the position of the tumor through analyzing the backscattering signals. The system setup is illustrated in Figure 13b where two prototypes of the antenna sensor are placed in the side by side Y direction at a distance of 250 mm. The distance from the phantom to the antenna is 58 mm. The PNA is connected to a PC with GPIB port for data collection and further processing of data. During the measurement, we have also inserted a cylindrical bowl containing material with dielectric properties similar to the pectoralis major muscle found under the breast tissue and repeated all the experiments. This inclusion caused the negligible change to the measured data. The modified delay and sum algorithm is designed to ignore multiple reflection signals by weighting all the delay and sum calculations with a correlation factor that rewards similarity between the delayed signals and penalizes signals from multiple reflections. They will exhibit lower correlation as the calculated delays will not account for the extra phase shift caused by a longer distance traveled by the multiple reflection signals.

## 7. Microwave Imaging Results and Discussions

For successful detection of a tumor, the antenna sensor performance is tested. For the measurements, the frequency range is considered from 3 to 11 GHz with M = 201 frequency points. From the measurement setup, the S-parameter (ϕl, *fm*) data in the frequency domain are recorded for l = 1, 2, …, L rotated positions, where L is 50. Thus the array records data for every 7.2° rotation, resulting in 50 observations. The observations are numbered 1 to 50 in the order they were recorded. Then it was observed that the even-numbered observation set is simply a 7.2° offset measurement of the odd-numbered observations. The S parameter matrix is thus split into two equal sized matrices of M × (L/2) size, named S-parameter-odd and S-parameter-even. Then the Γ(*φ_n_*, *f_m_*) matrix is generated by subtracting S-parameter-even from S-parameter-odd, where m and n denote the angular spot of every single spin. Here *m* = 1, 2, …, M and *n* = 1, 2, …, L/2. After collecting the data, the image of the interior of the phantom is constructed by post-processing. The conversion of the reflection coefficient to the time domain from frequency domain is done by using the inverse discrete Fourier transform:

(4) $$\begin{array}{cc} S(\varphi_{n},t_{k}) & =\exp\{[D_{k\times m}]\}\times \Gamma (\varphi_{n},f_{m})\\ & =\left[\begin{array}{ccc} S(\phi_{1},t_{1}) & \cdots & S(\phi_{N},t_{1})\\ \vdots & \ddots & \vdots \\ S(\phi_{1},t_{m}) & \cdots & S(\phi_{N},t_{k}) \end{array}\right] \end{array}$$

where:

(5) $$f_{m}=f_{1}+(m-1)(f_{h}-1)/(M-1)\;$$

(6) $$\Gamma (\varphi_{n},f_{m})=\left[\begin{array}{ccc} \Gamma (\phi_{1},f_{1}) & \cdots & \Gamma (\phi_{N},f_{1})\\ \vdots & \ddots & \vdots \\ \Gamma (\phi_{1},f_{m}) & \cdots & \Gamma (\phi_{N},f_{M}) \end{array}\right]\;$$

(7) $$[D_{k\times }{}_{m}]=\left[\begin{array}{ccc} j\omega_{1}t_{1} & \cdots & j\omega_{m}t_{1}\\ \vdots & \ddots & \vdots \\ j\omega_{1}t_{m} & \cdots & j\omega_{m}t_{k} \end{array}\right]\;$$

**Figure 14:**
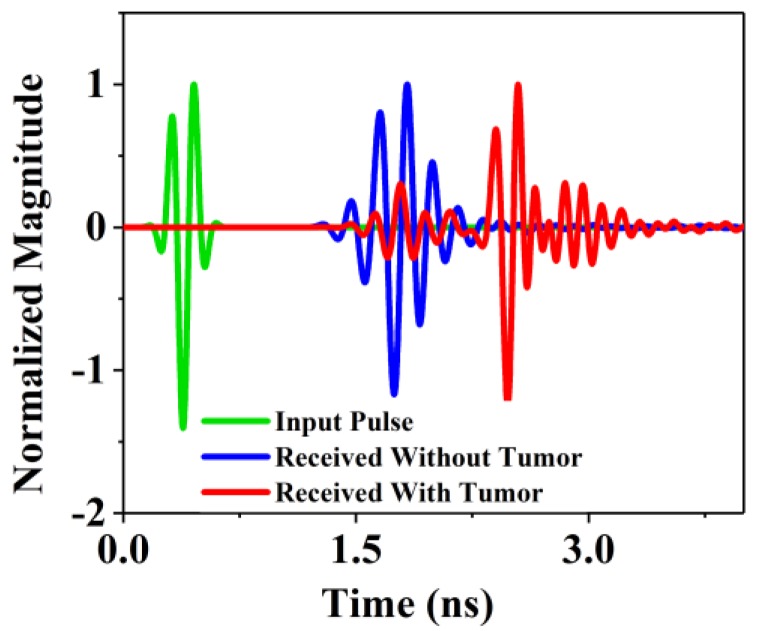
Normalized magnitude with and without the presence of a tumor inside the breast phantom.

**Figure 15:**
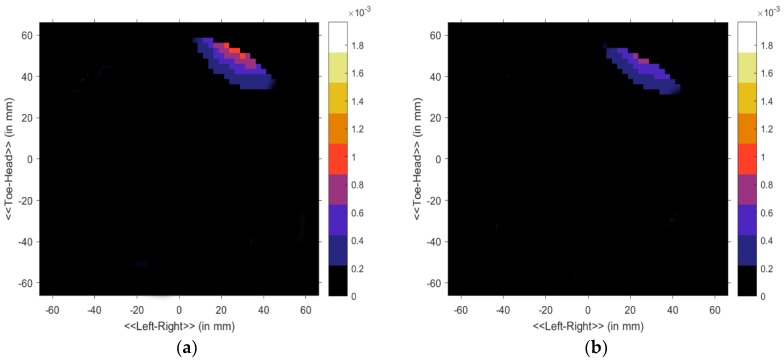
Microwave imaging results of breast phantom. (**a**) Without skin layer with tumor and; (**b**) with skin layer with a tumor inside the phantom.

Here *ω_m_* denotes the angular velocity and *k* denotes the equal distant points. Subsequently, the data in the S matrix was processed using the Delay-Multiply-and-Sum (DMAS) algorithm for the clear reconstruction of the image [45]. The sharp image of the internal construction of the breast phantom is shown in the processed image. Figure 14 shows the normalized magnitude of the proposed antenna system for two scenarios, without the presence of a tumor inside the phantom and with the tumor. A significant difference is noticed in the received pulses that the received pulse delay is not equal for both scenarios. This indicates the presence of unwanted cells inside the phantom for which the scattered signals cause delay at the receiving end. Since we are using cylindrically symmetric homogeneous phantom (excluding the cyst) and the phantom is placed at the center of the rotation axis, thus the skin reflections are nearly identical for all observations and any discrepancies between the even and odd sets of data must be obtained due to scattered signals from the internal structure of the phantom. The addition of uniform lossy material as the skin would mainly reduce the magnitude of the scattered field. The phase shift caused by the thin layer of skin around the phantom is similar to the shift caused the phantom material as the real part of their dielectric constants are comparable. Thus a modified delay and sum algorithm were used as it mainly relies on the phase of the scattered signal. In summary, the contribution of this skin material would be practically constant with respect to the rotation. The image generated from the backscattering analysis is shown in Figure 15a,b. Both the antennas act as a transducer. After analyzing the data with the presence of tumor it is clear that the tumor is detected at the (40, 50) position in the phantom position in Figure 15a,b. In Figure 15a, the tumor is identified with more red color concentration in the without skin layer scenario. On the other hand in Figure 15b, the tumor is identified as a blur because of the skin effect. The presence of a tumor is identified as deep red due to the higher return loss of the tumor. The signal cross through the normal breast tissue has no significant variation of the scattered signal while the noticeable change is observed in the second scenario that ensures the presence of unwanted cells. The proposed antenna and imaging system performance are better than those of recently reported antipodal Vivaldi antenna and imaging systems. Some of the systems are the only simulation-based [15] and some others have scarce imaging performance [17] or insufficient post-processing [5,35].

The proposed antenna has been compared with other antennas in Table 4. Comparing the proposed antenna with others, it is observed that, this antenna can be an eligible microwave imaging sensor candidate in terms of compact size, broad frequency of operation, high gain, directional radiation with high fidelity factor, homogenous phantom and imaging system development with tumor detection which are highly desired specifications.

## 8. Conclusions

A complete and compact medical imaging system with a BSAVA sensor is developed. The design and prototyping of a compact modified Vivaldi antenna sensor are done for testing the performance of the antenna sensor in microwave imaging systems. After the proper characterization, both the frequency domain and time domain behavior of the antenna sensor is verified. The microwave antenna sensor has a fractional bandwidth of 114% from 3.01 to 11 GHz with directional radiation pattern, higher gain, and efficiency. For enhancing the bandwidth, gain, directional radiation pattern and higher efficiency the different antenna sensor parameters are optimized including slots on both patch and ground. The antenna sensor performance of both frequency domain, time domain scenarios and reasonable Q factor with NFD are also investigated. After the fabrication of a realistic homogenous breast phantom, two prototypes are used for breast phantom measurement systems for detecting a tumor inside a homogenous breast phantom. The system is developed using a moving mechanical setup rotated by a stepper motor and controlled by Arduino-Uno for collecting data in the breast phantom. A customized system with a transducer, data collection model, and image reconstruction method has been developed based on MATLAB. There is a remarkable variation of the scattered signal is the key point to locate the unwanted cells inside the human breast. After proper analysis of these changes, the decision can be made that the proposed system can be used in localizing tumor cells among the healthy tissue and the system can be used as an early detection approach for unwanted tumors.

## Figures and Tables

**Table 1:** Different adjusted design parameters.

Parameters	mm	Parameters	mm
*L*	40	*G_S_*	8
*W*	40	*G_t_*	35
*P_W_*	9	*G_f_*	25.5
*P_L_*	12	*G_d_*	20
*G_W_*	9	*W_f_*	1.4
*G_L_*	12	*T*_1_, *T*_2_, *T*_3_, *T*_4_, *T*_5_, *T*_6_	0.5
*h*	1.6		

**Table 2:** Comparison of results on different modifications.

Different Modifications	Operating Bandwidth (GHz)	Peak Realized Gain (dBi)
Basic	3.01–5.31	5.62
Patch slotted	2.90–5.12	6.33
Ground slotted	3.01–5.36	5.54
Proposed	3.01–11	7.06

**Table 3:** List of materials and mixing ratios used for homogeneous phantom.

Material	Quantity				Purpose
	Fatty Phantom	Tumor	Skin	Muscle	
Distilled water	420 mL	420 mL	80 mL	420 mL	Solvent
Polyethylene powder	500 g	43 g	-	100 g	Modifying electrical permittivity
Agar	20 g	20 g	5.88 g	20 g	Mechanical strength
NaCl	2.3 g	28.3 g	-	22.5 g	Modifying electrical conductivity
Xanthan gum	6.25 g	6.25 g	7 g	6.25	Thickener
Sodium dehydroacetate monohydrate	0.25 g	0.25 g		0.25	Preservative
Safflower oil	-	-	14 mL		Modification of electric conductivity
Propylene glycol	-	-	7 g		Modification of electric conductivity
Formalin	-	-	0.3 mL		Rising melting temperature of agar-gelatin and phantom stabilizing
Detergent	-	-	0.3 g		Surfactant

**Table 4:** Comparison between different antennas with the proposed BSAVA.

Ref. No.	Size (mm^2^) λ_0_ × λ_0_	Type of Antenna	Operating Freq. (GHz)	Gain (dBi)	Applications	Observations
[5]	63 × 51 0.52 × 0.42	Vivaldi	2.5–8.5	8.5	Microwave breast imaging	Comparative large dimension, low gain at a lower frequency and no measured imaging results
[17]	75 × 75 0.125 × 0.125	Vivaldi	0.5–4.5	7	Microwave radar imaging	Unidirectional radiation with large dimension and imaging results are not characterized
[39]	88 × 75 0.4λ × 0.5λ	Vivaldi	1.54–7	8.5	Microwave breast imaging	Complex feed structure with large size, but directional radiation and good gain
[46]	100 × 53.19 0.66 × 0.35	Antipodal Vivaldi	2–27	7	Microwave imaging	Unidirectional properties obtained. Dimension is very large. Unavailability of the measured imaging results
[47]	110.3 × 100	Lens-loaded Vivaldi	1–14	<3 dB at lower freq. (2 GHz)	Microwave imaging	Large dimension. Low Gain at lower frequency with omnidirectional radiation
Proposed	40 × 40 0.40 × 0.40	Bbalanced slotted antipodal Vivaldi Antenna	3.01–11	7.1	Microwave breast imaging	Compact dimension with broad impedance bandwidth, a directional radiation pattern with high gain, high fidelity factor, a homogenous phantom with skin layer and imaging system development with tumor detection

**Figure A2:**
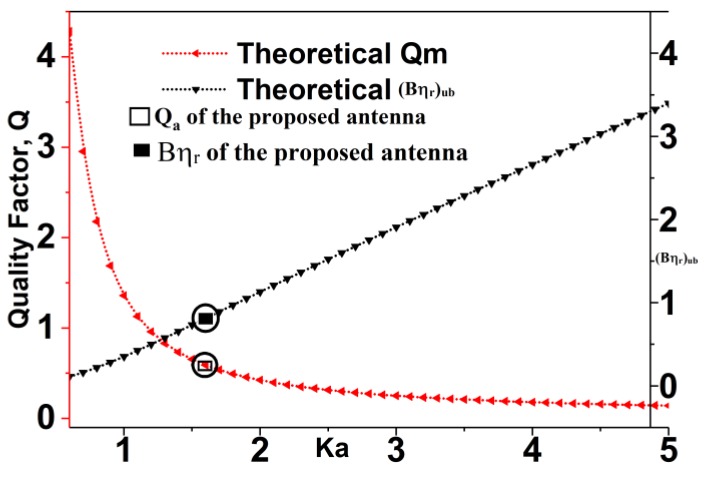
Theoretical limits and calculated Qa and (Bηr)_ub_.

**Figure A3:**
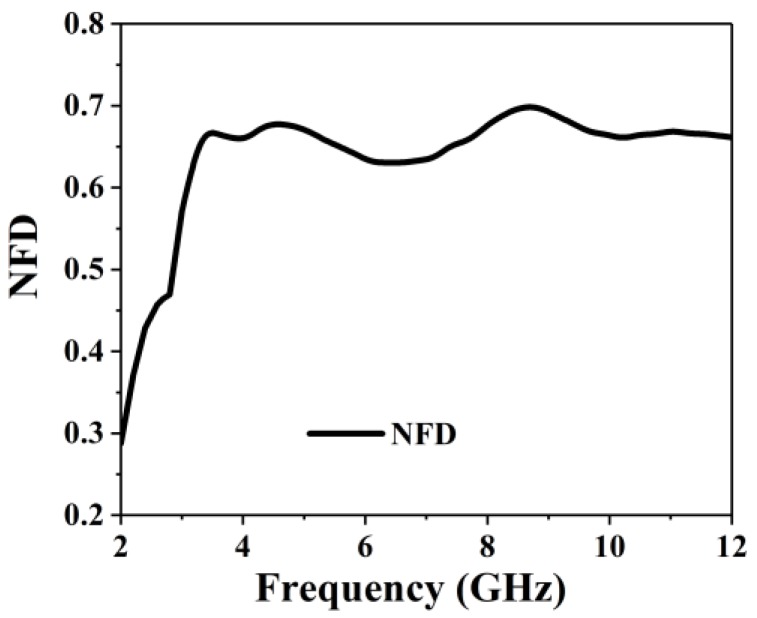
Simulated NFD of the proposed antenna.

## Appendix A. Time Domain Performance

The time domain performance of the BSAVA at three different scenarios of face to face, side by side X and side by side Y at 250 mm distant is presented in Figure A1a–c. In face to face operation, the received waveforms are the same as the transmitted pulses. Because of being a highly directive antenna, the antenna can send short pulse with very low distortion at the minimum timing. For side by side X and side by side Y scenarios the received waveforms slightly different from the actual waveforms. For directional radiation the side radiation slightly distorted and the mismatch is observed between the transmitted and received signals. A key factor is known as the fidelity factor (FF) [48] is also calculated to validate the correspondence between the transmitter (TX) and received (RX) signals:

(A1) $$F=\max\frac{\int_{-\infty }^{+\infty }m\left(t\right)n\left(t-\tau \right)dt}{\sqrt{\int_{-\infty }^{+\infty }\left|m{\left(t\right)}^{2}\right|dt\int_{-\infty }^{+\infty }\left|n{\left(t\right)}^{2}\right|dt}}$$

where, *m*(*t*) and *n*(*t*) represents the TX and RX signals, respectively. The fidelity factor is calculated in MATLAB by using this equation. The fidelity factor is 0.94, 0.85 and 0.98 for the face to face, side by side X and side by side Y position, respectively. The value to fidelity factor of the proposed BSAVA is higher than that of other recently published Vivaldi antennas. The higher value of fidelity factor ensures lower distortion of the transmitted signal that is a prerequisite for microwave imaging. The signal phase distortion is represented by the group delay. The group delay is measured from the negative derivatives of the phase response against frequency and presented in Figure A1d. Among the three set up the group delay of side by side, Y is less than both the face to face and side by side X. This is almost constant over the frequency, as a result, this setup is recommended for microwave imaging systems.

## Appendix B. NFD and Q Factor Analysis

With respect to the physical dimension of the antenna, the minimum quality factor can be achieved by using the equation $Q_{\mathrm{lb}}=\;\eta Q$ [49], where, $k=\frac{2\pi }{\lambda }$ and a denotes minimum sphere radius enclosed to the antenna. The higher bound of the radiation efficiency is:

(A2) $${\left(B\eta r\right)}_{\mathrm{ub}}=\frac{1}{\sqrt{2}}{\left[\frac{1}{\mathrm{ka}}+\frac{1}{k^{3}a^{3}}\right]}^{-1}$$

The $Q_{m}$ and (Bηr)_ub_ for different theoretical values of ka is shown in Figure A2. The antenna calculated value of ka = 1.78 for the 1st resonant frequency. From the ideal curve, it is noticed that the minimum limit of Q_m_ is 0.59 and (Bηr)_ub_ = 0.81.

The quality factor of the prototype is estimated through:

(A3) $$Q_{a}=\frac{2\sqrt{\beta }}{B}$$

where:

(A4) $$\sqrt{\beta }=\frac{s-1}{2\sqrt{s}}\leq 1$$

Here s = 2 is considered as the maximum accepted VSWR. The achieved Qa of the anticipated antenna is 0.56 and (Bηr)_ub_ = 0.80 which indicate that the theoretical and calculated value is very close. This verifies that the antenna design is optimum.

The NFD can be computed by using the formula presented in [50]. The NFD factor is the proportion of the power radiated inside the front side (Pf) and through the surface of the phantom (PT):

(A5) $$\mathrm{NFD}=\frac{\mathrm{Pf}}{\mathrm{PT}}$$

Figure A3 illustrates the NFD of the proposed antenna with a breast phantom. It is observed that about 67% of the total power is emitted through the front side of the breast tissue.
